# Naukan ethnobotany in post-Soviet times: lost edibles and new medicinals

**DOI:** 10.1186/s13002-017-0188-1

**Published:** 2017-11-17

**Authors:** Kevin A. Jernigan, Olga S. Belichenko, Valeria B. Kolosova, Darlene J. Orr

**Affiliations:** 1 University of Alaska Fairbanks, 505 N Chandalar Dr, Fairbanks, AK 99775 USA; 2 European University at St. Petersburg, Gagarinskaya ul., 3a, St Petersburg, Leningrad Oblast 191187 Russia; 3 Institute of Linguistic Studies, Tuchkov pereulok 9, Saint-Petersburg, 199053 Russia

**Keywords:** Ethnobotany, Ethnomedicine, Traditional knowledge, Wild edibles, Medicinal plants, Chukotka, The Naukan Yupik

## Abstract

**Background:**

This study focuses on health-related plant use among speakers of the critically endangered Naukan language (Inuit-Yupik-Unangan family) in the Russian Far East. The Naukan people were forced, in 1958, under Soviet consolidation, to move from their original settlement on Cape Dezhnev, leading to significant changes in spiritual worldview, subsistence, social structure, and language proficiency in the years that followed. Here, we focus on changes that elders report in their edible, medicinal, and spiritual uses of local plant species since their childhood.

**Methods:**

The authors worked from 2014 to 2016 in the villages of Lavrentiya, Lorino, and Uelen, in the Chukotskiy district of the Chukotka autonomous region, directly adjacent to the Bering Strait. We conducted structured interviews, using an oral history approach, along with participant observation and collection of voucher specimens from the local arctic tundra. Those with Naukan names and uses represent 42 species in 25 families.

**Results:**

Participants reported a decrease of 13% in the number of edible species that people currently harvest, from what they recall harvesting in their youth. On the other hand, the number of local species considered to be medicinal has actually increased by 225%. Current and past Naukan medicinal practices diverge in some notable ways from those of neighboring societies on the Alaskan side of the Bering Strait. Most of the spiritual significance of local plants species is remembered by only a few elders.

**Conclusions:**

Naukan elders explained the large increase in use of medicinal plants by noting that their original concept of medicine emphasized prevention and that illnesses were often assigned a spiritual rather than physical cause. Increased integration with ethnic Russians after moving from Naukan led to the adoption not only of new plant uses, but also of an entirely different, more naturalistic way of viewing illness and treatment.

## Background

### Introduction

**Fig. 1:**
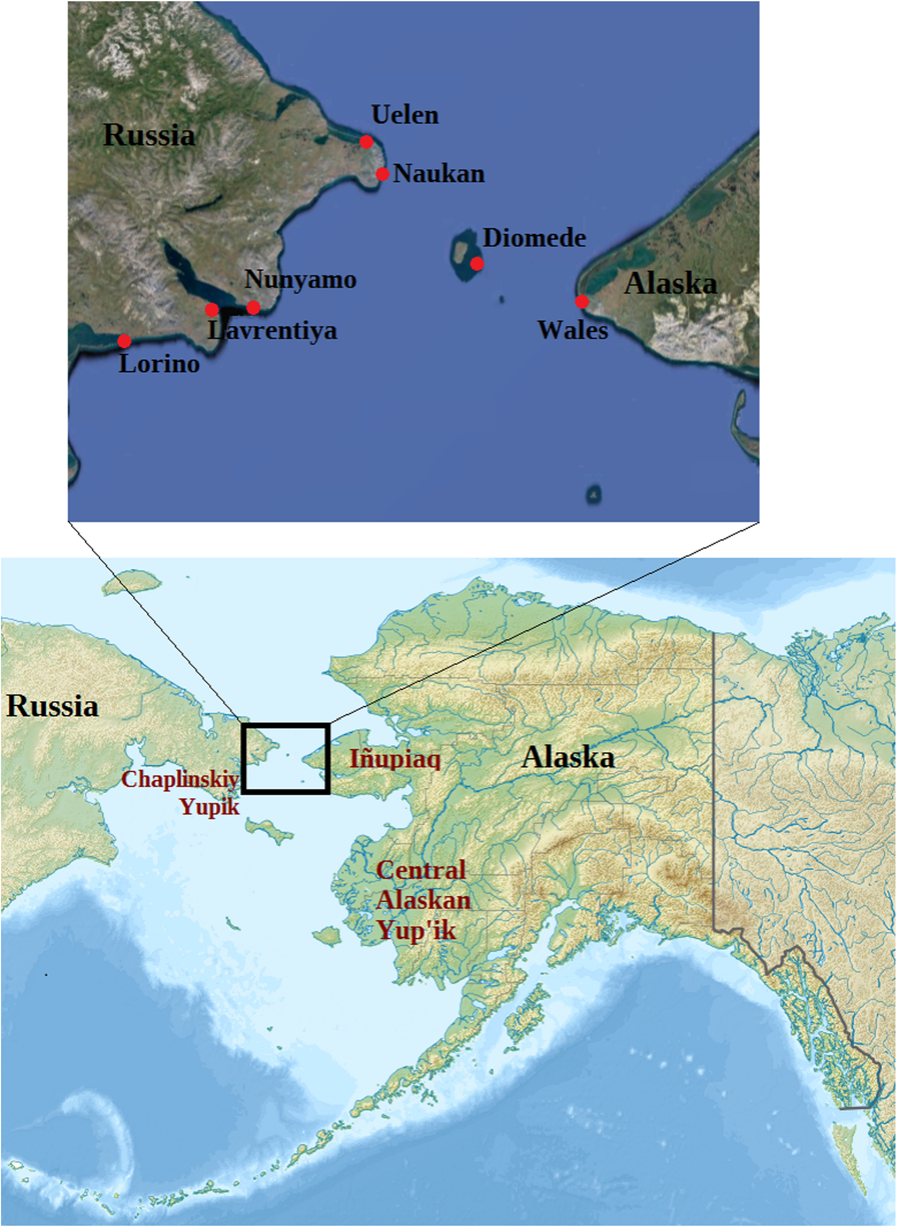
Map of the study area and region

Despite the harsh arctic climate of Chukotka, a region in the far northeast of Russia (Fig. 1), plants have played an important role in the subsistence and medical practices of local native peoples [1–3]. This is true not only in the short summer growing season, but also throughout the year, due to storage techniques including drying, fermenting, and soaking in animal fat [3, 4].

The earliest glimpses of this region’s ethnobotany can be found in Frans Reinhold Kjellman’s work with the coastal Chukchi in 1878–1879 [5, 6]. Bogoraz [7, 8] also provided some information on plant use in his more general ethnographic accounts. The Soviet period brought some more focused work on local plant uses. Notable studies include Tixomirov’s [9] and Menovshchikov’s [10] description of Chaplinskiy Yupik plant uses and Sokolova’s [11] and Mimykg Avtonova’s [12] work on Chukchi ethnobotanical practices. However, there are important gaps in the knowledge [2, 3] of this region, and documenting the continuing importance of plants to these societies in the post-Soviet context [1, 13] is especially urgent. One notable recent study [14] has compared attitudes toward edible fungi among the Siberian Yupik (Chaplinskiy and Naukan) and Chukchi in Chukotka and the Iñupiat on Alaska’s Seward Peninsula.

In particular, there has been very little ethnobotanical work done with speakers of Naukan (Inuit-Yupik-Unangan family) [15]. Dobrieva et al. [16] listed names for plants in their Naukan dictionary. Mimykg Avtonova [12] and Menovshchikov [10] documented some uses of edible species within larger studies. In the broader ethnomedical perspective, Bogoraz [17] and Tein et al. [18] explained the spiritual beliefs and healing practices of the Chaplinskiy Yupik and Naukan in the first half of the twentieth century. However, this article represents the first published work focusing specifically on ethnobotany of the Naukan people.

### History of the Naukan

**Fig. 2:**
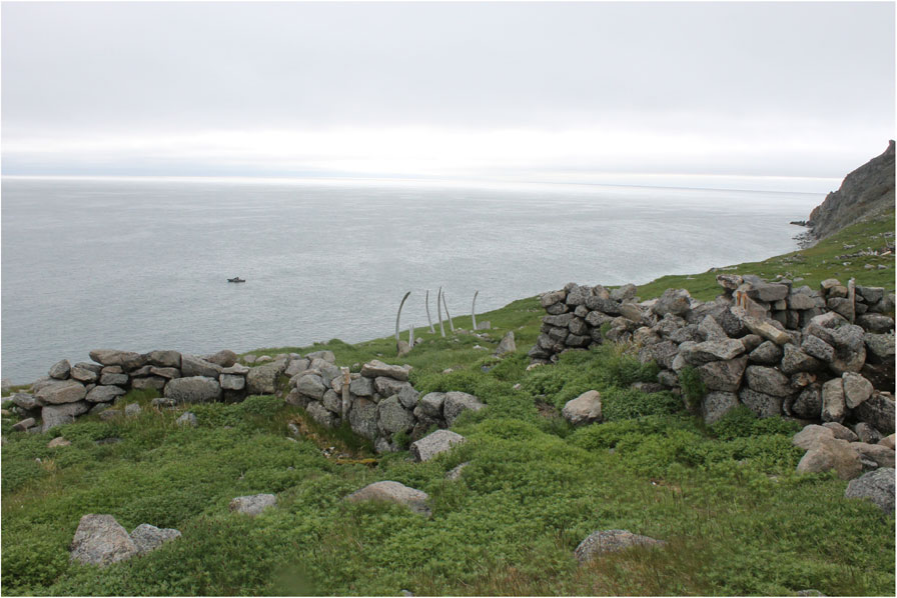
The old village site of Naukan

The village of Naukan (originally called Nevuqaq) (Fig. 2) was built on Cape Dezhnev, at the extreme eastern end of Eurasia. Subsistence practices focused on hunting sea mammals including the gray whale (*Eschrichtius robustus*), walrus (*Odobenus rosmarus*), spotted seal (*Phoca largha*), and bearded seal (*Erignathus barbatus*). This was supplemented by hunting of land mammals and gathering of plants and smaller marine organisms. During the Russian Imperial and early Soviet period, the site served as an important center for commercial and cultural exchange between the Chukchi on the Russian side and the Iñupiat on the Alaskan side of the Bering Strait. Intermarriages were common between the people of Naukan and the islands of Big and Little Diomede, in the Russian and US territories, respectively [16].

In 1958, the Soviet government closed Naukan as part of a larger program of consolidation of local population centers, and residents were forced to move to the neighboring Chukchi villages of Nunyamo and Uelen. Nunyamo, in turn, was closed in 1977, and local people moved from there to the villages of Lavrentiya and Lorino, where most reside today.

Following relocation, the Naukan people and their culture experienced significant changes in spiritual worldview, subsistence practices, social structure, and language proficiency. Waves of military and civilian migrants from other parts of the Soviet Union also contributed to these broad changes through direct personal interaction, including intermarriages. Although Naukan people did not experience the acculturative influences of missionary activity that were widespread on the Alaskan side of the Bering Strait, shamans were persecuted and the accompanying spiritual practices were greatly challenged by the dominance of materialism under Soviet rule [19].

The period immediately after the collapse of the Soviet Union, particularly the second half of the 1990s, was also quite difficult for the study region, as economic support from the central government was withdrawn. One result was the revival of sea mammal hunting brigades to meet the nutritional needs of local people, who were left with very little food in village stores [20]. The economic situation has improved somewhat in the last decade. Currently, the Naukan language is considered critically endangered [21], a designation meaning that only some members of the oldest generation speak it.

## Methods

The authors conducted work from 2014 to 2016, principally in the villages of Lavrentiya, Lorino, and Uelen, in the Chukotskiy district of Chukotka, Russia (Fig. 1). The study also included a few interviews with Naukan speakers in the regional capital of Anadyr and two in the Alaskan towns of Nome and Kotzebue. All study sites have arctic tundra vegetation. The village of Naukan and the area immediately around it are dominated by noncarbonate mountain complex tundra with nearby regions of low shrub and wetland tundra [22] around neighboring villages.

The research was part of a larger study comparing the plant traditions of cultures on the Alaskan and Russian sides of the Bering Strait, funded by NSF grant number 1304612 [3]. The goal of the project was to document and compare edible, medicinal, and spiritual plant use among the Naukan and Chukchi peoples of Chukotka, Russia, and the Central Alaskan Yup’ik. Human subjects approval was obtained from the University of Alaska, Fairbanks Institutional Review Board (IRB), prior to beginning work. The study conformed to the American Anthropological Association’s ethical guidelines [23], and prior informed consent was given by all participants.

We began in each village by meeting with local community members to answer questions, discuss the research goals, and recruit potential participants. Since Naukan is a critically endangered language, we attempted to interview as many speakers as possible, rather than aiming for a representative sample. Dorais [24] estimated the number of speakers as 60. However, our detailed discussions with elders, asking them to freelist every speaker they know, yielded a more conservative figure of 29 individuals. We succeeded in interviewing 21 (76%) full speakers and an additional seven partial speakers.

Interviews focused on local plants used presently or in the past for nutritional or health-related purposes. For each species, we asked participants to freelist edible, medicinal, and spiritual uses as well as times and methods of harvest, preparation, and storage. For every use given, we also asked the following: (a) if it was practiced during the participant’s youth and (b) if it is currently practiced.

A number of researchers [25–27] have found oral history to be a useful approach in achieving a time depth in ethnobotanical studies, particularly when there are no suitable past studies or archival records to use for comparison. To help mitigate potential weaknesses of oral histories [28, 29], including selective or inaccurate memories of past events and practices, researchers often combine this method with other approaches. For example, oral history has been corroborated with linguistic evidence [30, 31] and comparative ethnography [32–34]. For the current analysis, we use evidence from Soviet popular literature [35–38] to support claimed borrowings. Reports from neighboring indigenous groups [39–41] lend credibility to accounts of discontinued uses.

Naukan participants gave a name or use for 39 local plant species, in 22 botanical families and three algae species in three families. Vouchers of these are stored in the herbarium of the Komarov Botanical Institute in St. Petersburg, Russia, and were determined there with the help of botanist Vladimir Razzhivin. We compiled a list of 42 Naukan folk genera [42], including nine whose botanical identity is not still remembered. All but one of these are monotypic. In this article, we focus on those species that study participants considered to be important for human health and nutrition.

## Results and discussion

Table 1 includes those Naukan names whose botanical identity is known and summarizes their uses. Although the major focus of this article is on plants with health-related uses, we considered it important to list others here as well, due to the dearth of published literature [15] on Naukan ethnobotany. Algae species are included, since local people group these with plants [1, 2, 43] in their folk taxonomy. We do not consider fungi, however, as Yamin-Pasternak [14] has already made a thorough treatment of that subject. Russian names are those used by local people for these species. The same names may refer to different species in other parts of Russia.

**Table 1:** Summary of Naukan plant use

Species	Vouchers	Naukan name	Russian name	Current UC	Current UV	Past UC	Past UV
Alariaceae							
*Alaria marginata* Postels & Ruprecht	KAJR74	*elquaq*	мopcкaя кaпуcтa	Edible, medicinal	2.1	Edible	1.9
Amaryllidaceae							
*Allium shoenoprasum* L.	KAJR104	*mayughlak*	дикий лук	Edible	1.5	Edible	1.17
Apiaceae							
*Angelica gmelinii* (DC.) Pimenov	KAJR57	*ikiituk*	–	Spiritual, medicinal	2.01	Spiritual, medicinal	1.86
Asreraceae							
*Artemisia tilesii* Ledeb.	KAJR35	*saayyge*	пoлынь	Medicinal, spiritual, insect repellant	0.9	Spiritual, hygiene	0.19
*Petasites frigidus* (L.) Fr.	KAJR7	*llamquq*	мaть-и-мaчexa	Edible, medicinal	0.75	Edible	1.12
*Taraxacum macilentum* Dahlst.	KAJR38	*supughayaghqaq*	oдувaнчик	–	0	–	0
Betulaceae							
*Betula* sp.	KAJR53	*gulgiile*	бepёзa	Kindling	0.14	Kindling, containers	0.29
Boraginaceae							
*Myosotis alpestris* F.W.Schmidt	KAJR8	*sunganguaq*	нeзaбудкa	–	0	–	0
Caryophyllaceae							
*Silene uralensis* subsp. *apetala* (L.) Bocquet	–	*awataghpaguaq*	–	–	0	Play	0.22
Crassulaceae							
*Rhodiola integrifolia* Raf.	KAJR18	*saqlak*	poдиoлa poзoвaя	Edible, medicinal	1.88	Edible	1.44
Cyperaceae							
*Eriophorum angustifolium* Honck.	KAJR17	–	пушицa	Edible	0.08	Spiritual	0.08
Ericaceae							
*Arctous alpina* (L.) Nied.	KAJR28	*alaglukaq*	вoлчья ягoдa	–	0	Play	0.31
*Cassiope tetragona* (L.) D.Don	KAJR96	–	–	Tinder	0.29	Tinder	0.29
*Empetrum nigrum* L.	KAJR22	*akuvilqaq*	шикшa	Edible, medicinal	2.2	Edible	1.4
*Rhododendron tomentosum* Harmaja	KAJR11	–	бaгульник	Medicinal	0.31	–	0
*Vaccinium uliginosum* L.	KAJR24	*sughhaq*	гoлубикa	Edible	1.5	Edible	1.33
*Vaccinium vitis-idaea* L.	KAJR21	*mesutaq*	бpуcникa	Edible, medicinal	2.56	Edible	0.81
Fabaceae							
*Hedysarum hedysaroides* (L.) Schinz & Thell.	KAJR29	*unataq*	–	Edible	0.38	Edible	0.62
Fucaceae							
*Fucus evanescens* C.Agardh	KAJR33	–	мopcкaя кaпуcтa	Edible	0.19		0
Laminariaceae							
*Laminaria saccharina* (L.) Lamouroux	KAJR73	*nuvakataq*	мopcкaя кaпуcтa	Edible, medicinal	0.89	Edible	0.7
Montiaceae							
*Claytonia acutifolia* Pall. ex Schult.	KAJR45	*kegtaq*	–	–	0	Edible	0.27
*Claytonia tuberosa* Pall. ex Schult.	–	*ulqiq*	дикaя кapтoшкa	–	0	Edible	0.62
Onagraceae							
*Epilobium latifolium* L.	KAJR36	*wiawiagte*	ивaн-чaй	Edible, medicinal	1.87	Edible, medicinal	1.67
Orobanchaceae							
*Pedicularis verticillata* L.	KAJR46	*suaghraagte*	петушок	Edible	1	Edible	1.07
Plantaginaceae							
*Lagotis glauca* Gaertn.	KAJR43	*qungum neqenllaa*	–	–	0	Spiritual	0.13
Poaceae							
*Leymus mollis* (Trin.) Pilg.	KAJR37	–	–	–	0	Spiritual, insulation	0.63
Polygonaceae							
*Persicaria bistorta* (L.) Samp.	KAJR12	*neqenllaq*	гopeц	Edible	1.71	Edible	1.82
*Oxyria digyna* (L.) Hill	KAJR16	*quulngiq*	щaвeль, киcличник	Edible	1.93	Edible	1.33
*Polygonum tripterocarpum* A. Gray ex Rothr.	KAJR15	*qeghhyughaq*	щaвeль	Edible, medicinal	0.8	Edible, medicinal	0.5
*Rumex arcticus* Trautv.	KAJR31	*ngerngaq*	кoнcкий щaвeль	Edible	1.88	Edible	1.06
Ranunculaceae							
*Anemone sibirica* L.	KAJR50	*taquapik*	–	–	0	Edible	0.21
*Aconitum productum* Rchb.	KAJR4	*tekenguaq*	–	–	0	Play	0.61
Rosaceae							
*Dryas incisa* Juz.	KAJR52	*qateghyiaghhaq*	–	Edible	1	Edible	0.93
*Rubus chamaemorus* L.	KAJR19	*aqpik*	мopoшкa	Edible, medicinal	2.62	Edible	1.38
Salicaceae							
*Salix pulchra* Cham.	KAJR20	*ququngaq*	ивa	Edible, medicinal	1.21	Edible, medicinal	0.5
*Salix arctica* Pall.	KAJR154	*ququngaaghhaq*	кapликoвaя ивa	–	0	Edible	0.57
Saxifragaceae							
*Saxifraga nelsoniana* D. Don	KAJR9	*siiqnaq*	кaмнeлoмкa	Edible	1	Edible	1
*Saxifraga oppositifolia* L.	KAJR81	*neghyaq*	–	–	0	Edible	1
Sphagnaceae							
*Sphagnum squarrosum* Crome	KAJR44	*ungagaq*	мox	–	0	Hygiene, wicks	0.31

The focus here is on how the overall importance of species has changed over time. For the purpose of calculating use values (UVs) [44], we included only data from the 21 members of the oldest generation (ranging in age from 62 to 91), who remember the period before and immediately after the forced move from Naukan in 1958. However, interviews and participant observation with younger people did help to confirm whether the uses that elders described should be considered currently practiced or not.

Table 2 presents changes in gathering and use of edible plants from elders’ oral histories. Our extensive participant observation in summer field sessions from 2014 to 2016 also helps confirm which plants are still collected and prepared. The total number of edible species is 26. This is roughly comparable to what has been reported for neighboring societies (Fig. 1). For example, Kjellman’s pioneering work [5] with the coastal Chukchi yielded 23 food taxa, while Ainana and Zagrebin [1] recorded 26 edible species from the Chaplinskiy Yupik in the southern part of the Chukchi Peninsula. Young and Hall [45] listed 18 species in neighboring St. Lawrence Island. On the Alaskan mainland, where botanical biodiversity is a bit higher [46], Jones [47] described 33 edible plant species gathered by Iñupiat of the Kotzebue region and Ager and Ager [48] listed 34 on Nelson Island.

**Table 2:** Edible uses of plants

Species	Part	UVpres	UVpast	Maintained uses	Lost uses	Gained uses
Alariaceae						
*Alaria marginata* Postels & Ruprecht	Blades	2	1.9	Midrib eaten raw, whole blade dried and added to boiling meat	–	Midrib eaten fresh in salads
Amarillydaceae						
*Allium shoenoprasum* L.	Total	1.5	1.17			
	Leaves	1.33	1.17	Eaten fresh with meat	–	Salted to store
	Flowers	0.17	0	–	–	Salted to store
Asteraceae						
*Petasites frigidus* (L.) Fr.	Leaves	0.31	1.12	Gathered young, eaten with seal oil and meat	Stored in seal oil with *Saxifraga nelsoniana*	–
Crassulaceae						
*Rhodiola integrifolia* Raf.	Leaves, stems	1.44	1.44	Fermented, eaten later with meat and fat	Fermented juice was eaten with walrus chest	Eaten with sugar
Cyperaceae						
*Eriophorum angustifolium* Honck.	Tuber	0.08	0	–		Gathered from vole nests, eaten with seal oil
Ericaceae						
*Empetrum nigrum* L.	Berries	2	1.4	Eaten plain, with seal oil	Stored in seal oil	Jam, kompot, juice, wine, eaten with sugar, reindeer stomach
*Vaccinium uliginosum* L.	Berries	1.5	1.33	Eaten plain, with seal oil	Picked green, stored in seal oil	Jam, eaten with sugar
*Vaccinium vitis-idaea* L.	Berries	1.81	0.81	Eaten plain, with seal oil		Jam, kompot, mors, eaten with sugar, put in tea
Fabaceae						
*Hedysarum hedysaroides* (L.) Schinz & Thell.	Tuber	0.38	0.62	Harvested from ground in the fall, eaten with sea mammal fat	Harvested from vole caches, roots stored in oil for winter	–
Fucaceae						
*Fucus evanescens* C.Agardh	Receptacles	0.19	0	–	–	Eaten raw
Laminariaceae						
*Laminaria saccharina* (L.) Lamouroux	Blades	0.8	0.7	Eaten raw, dried and put in with boiling meat	–	Put in Russian style salad, pirogi
Montiaceae						
*Claytonia acutifolia* Pall. ex Schult.	Tuber	0	0.27	–	Boiled in liquid sea mammal fat and eaten with meat	–
*Claytonia tuberosa* Pall. ex Schult.	Tuber	0	0.62	–	Boiled in liquid sea mammal fat and eaten with meat	–
Onagraceae						
*Epilobium latifolium* L.	Leaves	1.4	1.2	Lightly boiled, pressed and stored moist, eaten with meat	–	Dried to store
Orobanchaceae						
*Pedicularis verticillata* L.	Total	1	1.07			
	Flowers	1	0.93	Eaten fresh on the tundra	–	Put in with fermenting *Rhodiola*
	Tuber	0	0.14	–	Eaten raw with seal oil	–
Polygonaceae						
*Persicaria bistorta* (L.)	Total	1.71	1.82			
	Leaves	1.06	0.94	Eaten with seal oil and dried meat	–	–
	Flowers	0.65	0.59	Eaten raw with seal oil and meat, children eat plain	–	–
	Tuber	0	0.29	–	Eaten raw with seal oil	
*Oxyria digyna* (L.) Hill	Total	1.93	1.33			
	Leaves	1.73	1	Eaten fresh with meat, seal oil and other greens	–	Mors, kompot, kisel, put in soup, eaten with vegetable oil
	Flowers	0.2	0.33	Eaten fresh with seal oil, plain	Drink made with water	Eaten with soup
*Polygonum tripterocarpum* A. Gray ex Rothr.	Leaves, stems	0.7	0.4	Eaten with seal oil	–	Eaten with seal oil and seal blood
*Rumex arcticus* Trautv.	Total	1.88	1.06			
	Leaves	1.69	0.81	Store in seal oil with *Saxifraga nelsoniana*, eaten with meat	–	Kompot, jam, put in soups, salads, fermented with *Rhodiola*
	flowers	0.19	0.25	Eaten with seal oil	Boiled in water to make drink	Kompot
Ranunculaceae						
*Anemone sibirica* L.	Aerial parts	0	0.21	–	Put in seal oil with *Saxifraga nelsoniana* for taste	–
Rosaceae						
*Dryas incisa* Juz.	Flowers	1	0.93	Eaten fresh with seal oil and dried meat	–	Eaten in Russian style salads
*Rubus chamaemorus* L.	Berries	1.81	1.38	Eaten plain, with seal oil, with other berries	Stored in seal oil, eaten with reindeer fat	Jam, kompot, syrup, eaten with sugar, *kefir*
Salicaceae						
*Salix pulchra* Cham.	Leaves	1.07	0.36	Put in fresh with boiling meat	Walrus skin cooked in juice	Dried or stored moist, eaten with boiled meat
*Salix arctica* Pall.	Total	0	0.57			
	Leaves	0	0.14	–	Eaten with seal oil and meat	–
	Flowers	0	0.43	–	Eaten with seal oil and meat, kids chewed new buds like gum	–
Saxifragaceae						
*Saxifraga nelsoniana* D. Don	Leaves	1	1	Leaves stored in seal oil, eaten with meat	–	–
*Saxifraga oppositifolia* L.	Flowers	0	1	–	Eaten with sea mammal oil and dried meat	–

As expected, the number of species gathered for food has decreased after integration with ethnic Russian neighbors and greater access to store-bought food. Of 26 total edibles, 19 (73%) have been retained from the days of elders’ youth. Two (8%) were adopted more recently, while five (19%) were discontinued. Some species in the latter group, such as *Claytonia tuberosa* Pall. ex Schult. and *Saxifraga oppositifolia* L., were readily available on the rocky mountainous slopes surrounding Naukan but are difficult to find in the relatively flatter terrain around the villages of Lavrentiya, Lorino, and Uelen where the Naukan people now live. Elders’ accounts suggest that some other practices, including gathering tubers of *Hedysarum hedysarioides* (L.) Schinz & Thell.from vole caches, were already becoming marginal even before people left Naukan.

Local accounts of the six discontinued edible species can be backed up using comparative ethnography. Here we cite reports of their use in neighboring societies and by other arctic peoples. The Central Alaskan Yup’ik [41] have harvested tubers of *Claytonia tuberosa* Pall. ex Schult. as well as leaves and flowers of the dwarf willow (*Salix*) species for food. Residents of King Island [39], in the Bering Sea, used to gather *Claytonia acutifolia* Pall. ex Schult. tubers and *Anemone sibirica* L. flowers. Egeland et al. [40] report that the Iñuit of Baffin Island eat the flowers of *Saxifraga oppositifolia* L.

For the 19 maintained edible species, there has also been an increase, in 14 cases (74%), in the ways they are prepared. Eleven of that group (79%) have acquired new uses, principally, from ethnic Russians. For example, wild greens are now added to soups such as *borsch* and the cabbage-based *shchi*, as well as eaten in the traditional manner with seal oil. Berries are now made into jams, kompot (stewed fruit), and mors (fruit drink). For three species, the new uses came from Chukchi neighbors [3]. For example, Naukan people traditionally fermented the leaves and stems of rose root (*Rhodiola integrifolia* Raf.) by itself. Now some people add the flowers of *Pedicularis verticillata* L. or the leaves of *Rumex arcticus* Trautv. as well.

Table 3 discusses past and present medicinal uses. Of 13 total species in this category, Naukan elders said nine (69%) are new additions since their youth and that none has been lost, leaving four (31%) with maintained use over that period. Although past studies [25, 49, 50] have demonstrated the importance that borrowed species and uses can play in local pharmacopeias, the Naukan elders’ claim to have previously used so few species medicinally and to have borrowed such a large proportion of the uses they currently practice can still be considered atypical [51, 52]. The Saami of northern Sweden [53] do provide another example where local plant species traditionally played a relatively minor part in healing practices compared to animal, mineral, and magical cures.

**Table 3:** Medicinal uses of plants

Genus	Species	Part	UVpres	UVpast	Maintained uses	Lost uses	Gained uses
Alariaceae							
*Alaria marginata* Postels & Ruprecht		Stem	0.1	0	–	–	*Contains iodine*, *good for general health* ^a^
Apiaceae							
*Angelica gmelinii* (DC.) Pimenov		Total	0.72	0.57			
		Root	0.57	0.57	Smoke good for coughs, asthma, aiding childbirth preventing illness in general, decoction used for headache	–	–
		Leaves	0.14	0	–	–	Eaten or used to make a steam bath to treat cold and cough
Asteraceae							
*Artemisia tilesii* Ledeb.		Leaves, stem	0.19	0	–	–	*Tea or steam bath for cough*, *steam bath for leg pain* ^b^
*Petasites frigidus* (L.) Fr.		Leaves	0.44	0	–	–	*Tea for cough and colds*, *steam bath for colds* ^b^
Crassulaceae							
*Rhodiola integrifolia* Raf.		Root	0.44	0	–	–	*Tincture is tonic* ^b^, helps sore throats; *cold water infusion for mood improvement and stomach problems* ^b^; warm water infusion used externally to strengthen hair
Ericaceae							
*Empetrum nigrum* L.		Berries	0.2	0	–	–	*Lower blood pressure* ^c^ and help diarrhea when eaten
*Rhododendron tomentosum* Harmaja		Leaves	0.31	0	–	–	*Expectorant tea for coughs and colds* ^b^
*Vaccinium vitis-idaea* L.		Total	0.75	0			
		Berries	0.38	0	–	–	*Colds* ^c^ and *fevers*; *high blood pressure* ^b^; heart health
		Leaves	0.38	0	–	–	*Tea for colds* ^c^, *high blood pressure*, *diuretic* ^b^, *steam bath as expectorant* ^c^
Laminariaceae							
*Laminaria saccharina* (L.) Lamouroux		Stem	0.09	0	–	–	*Contains iodine*, *good for general health* ^d^
Onagraceae							
*Epilobium latifolium* L.		Leaves	0.47	0.47	Good for diarrhea when eaten	–	–
Polygonaceae							
*Polygonum tripterocarpum* A. Gray ex Rothr.		Leaves	0.1	0.1	Good for diarrhea when eaten	–	–
Rosaceae							
*Rubus chamaemorus* L.		Sepals	0.81	0	–	–	*Tea for colds*, *cough*, *sore throat* ^b^, steam inhaled for asthma
Salicaceae							
*Salix pulchra* Cham.		Leaves	0.14	0.14	Good for diarrhea when eaten	–	–

^a^Barashkov [35] (This source deals with the biochemistry of algae species.)

^b^Krylov [36] (This source treats Soviet medical use and traditional uses of plant species in western Siberia.)

^c^Barnaulov et al. [38] (This source lists traditional Russian uses and biochemical properties of edible fruits and berries.)

^d^Trofimov [37] (This source deals with the biochemistry of algae species.)

Unfortunately, older literature on Naukan ethnobotany is scant [10], particularly for medicinal uses. So, we take another approach here to support elders’ reports of borrowings. Following other researchers [32–34], who have used comparative ethnography to help triangulate memory ethnography, we examine Soviet era popular literature on the medicinal and nutritional qualities of plants [35–38]. The idea is to test whether newcomers arriving in Chukotka from other parts of Russia, including professionals such as teachers, doctors, and health aids, could have brought new information on medicinal uses of local plants as Naukan elders described. We have italicized those uses in Table 3 where this comparative evidence (for the same or closely related species) supports the alleged borrowing.

Current and past Naukan medicinal plant use diverges in some notable ways from that of neighboring societies on the Alaskan side of the Bering Strait. Although the floras of both regions are very similar [54], the most salient medicinal species are different. For example, *Artemisia tilesii* Ledeb. and *Rhododendron tomentosum* Harmaja are the two most important and commonly mentioned medicinal plants among the Central Alaskan Yup’ik [41, 43] and Iñupiat [47, 55] of western Alaska. Although both species were cited by Naukan elders as medicinal, their use values of 0.28 (for *Artemisia tilesii* Ledeb.) and 0.31 (for *Rhododendron tomentosum* Harmaja) indicate that their importance in this pharmacopeia is minor. Also, Naukan elders claim that they did not use either species before Russian influence, while there are no similar claims of borrowing on the Alaskan side [41, 47]. By the same token, species of greater medicinal importance for the Naukan, such as *Angelica gmelinii* (DC.) Pimenov and *Rubus chamaemorus* L., are principally used for food among the Iñupiat [47] and Central Alaskan Yup’ik [41].

Naukan spiritual plant uses appear in Table 4. In current times, there is only really one species of any great spiritual importance, *Angelica gmelinii* (DC.) Pimenov. Its root is burned for a variety of ritual and medicinal purposes. Oral histories suggest this same plant was the most important spiritual species in the past as well, although there were others that very few elders remember now. The Central Alaskan Yup’ik people, in contrast, burn the strongly aromatic species *Rhododendron tomentosum* Harmaja for spiritual cleansing [41]. However, Oswalt [56] reported that Yup’ik people in the middle Kuskokwim region of Alaska remembered formerly burning the tops of dried *Angelica gmelinii* (DC.) Pimenov for smudging. More recently, Jernigan [41] reports a similar cultural memory from the village of Chevak, on the Bering Sea coast, of previous use of this same species for ceremonial cleansing during the *Nakaciuq* (Bladder festival).

**Table 4:** Spiritual uses of plants

Genus	Species	Part	UVpres	UVpast	Maintained uses	Lost uses	Gained uses
Apiaceae							
*Angelica gmelinii* (DC.) Pimenov		Root	1.29	1.29	Burned for spiritual purification of houses and individuals, burned or hung in house for luck, small piece eaten after first whale catch	–	–
Asteraceae							
*Artemisia tilesii* Ledeb.		Aerial parts	0.05	0.05	Hung up in house for good luck	–	–
Cyperaceae							
*Eriophorum angustifolium* Honck.		Aerial parts	0	0.08	–	Thrown into sea as offering to ensure future harvests	–
Plantaginaceae							
*Lagotis glauca* Gaertn.		Flowers	0	0.13	–	Associated with the dead	–
Poaceae							
*Leymus mollis* (Trin.) Pilg.		Leaves	0	0.18	–	Spiritual significance for funerals	–

It is worth noting that our findings about one particular species of spiritual significance to the Naukan, *Lagotis glauca* Gaertn., conflict with some earlier published accounts. Both Menovshchikov [10] and Ainana and Zagrebin [1] wrote that this species is called *ngerngaq* in Naukan and is stored with *Saxifraga nelsoniana* D. Don leaves in seal oil to eat during the winter. However, all participants in this study assigned the name *ngerngaq* and its edible use to *Rumex arcticus* Trautv. No one we interviewed considered *Lagotis glauca* Gaertn. to be a food, and most did not know a name or significance. A few elders independently gave the name *qungum neqenllaa*—“dead man’s bistort.” The name stems from a resemblance to the local edible species *Persicaria bistorta* (L.) Samp. and comes from the fact that this plant was considered to be food for the dead. The discrepancy with earlier published literature can be explained by noting that Menovshchikov [10] and Ainana and Zagrebin [1] did most of their fieldwork with speakers of the related Chaplinskiy Yupik language, rather than in the Naukan area.

## Conclusion

Testimony of Naukan elders indicates a decrease in the number of species harvested for food over the last 60 years. However, there has been an increase in the ways that edible plants are prepared due to the influence of Russian and Chukchi neighbors. Knowledge of spiritual species has also shown significant decline.

The most surprising result of this research is the direction of change in medicinal plant use. The Naukan present an interesting case where acculturative forces appear to have significantly expanded the botanical pharmacopeia through the borrowing of ethnic Russian traditions. Older Naukan participants often said that their original concept of medicine emphasized prevention. For example, the leaves of willows (*Salix pulchra* Cham.) and willow herb (*Epilobium latifolium L.*) aid the digestive system and help prevent stomach upset when they are eaten as part of a meal. This traditional emphasis on staying healthy reflects findings by researchers working in some other parts of the arctic as well. For instance, Hakkarainen [57] has observed that the reindeer-herding Chukchi have tended to use plants for maintaining health rather than curing sickness. Black et al. [58] report that preventative medicine is the most important category for the Iñuit of Qikiqtaaluk, Nunavut.

Naukan elders also emphasized that, when people became sick in the old days, they often assigned a spiritual cause. So that meant the cure also had to be spiritual rather than physical in nature. Bogoraz’s [7, 8] classic ethnography with the neighboring Chukchi also mentions a dearth of medicinal plant usage, stating that cures were largely spiritual. In contrast to many other parts of the world [59, 60], Naukan elders reported that they did not rely on psychoactive plants to achieve altered states of consciousness. According to native scholar Tein et al. [18], Naukan shamans established relationships with helper spirits in visions or dreams. The spirits, in turn, would teach the healers special songs that would allow the latter to make contact in the future. The shamans also healed the sick with the help of these spirits and with symbolic acts like changing the name of a sick child or blowing an illness toward the door of a dwelling. So, it is clear that increased integration with ethnic Russians after moving from Naukan led to the adoption not only of new plant uses, but also of an entirely different, more naturalistic way of viewing illness and healing.

The authors are currently carrying out research on the ethnobotanical traditions of the neighboring Chukchi and the Central Alaskan Yup’ik to expand the discussion of the plant traditions of the Bering Strait region. Future work could also expand the region of study to other adjacent locations such as Little Diomede and the Seward Peninsula which both had a high degree of historical contact [16] with the Naukan people.

## Abbreviations

IRB: Institutional Review Board
UC: Use category
UV: Use value
